# Elucidating the Changes in Metabolites and Physicochemical Quality of Greater Amberjack (*Seriola dumerili*) During Wet Aging

**DOI:** 10.3390/foods15183311

**Published:** 2026-09-18

**Authors:** Di Wang, Ya Wei, Chunsheng Li, Gang Yu, Jianwei Cen, Yanyan Wu, Zhenhua Ma, Xue Zhao, Shan Huang, Yongqiang Zhao

**Affiliations:** 1 Key Laboratory of Aquatic Product Processing, Ministry of Agriculture and Rural Affairs, South China Sea Fisheries Research Institute, Chinese Academy of Fishery Sciences, Guangzhou 510300, China; wangdi@scsfri.ac.cn (D.W.);; 2 Key Laboratory of Efficient Utilization and Processing of Marine Fishery Resources of Hainan Province, Sanya Tropical Fisheries Research Institute, Sanya 572426, China; 3 College of Food Science and Engineering, Ocean University of China, Qingdao 266000, China

**Keywords:** greater amberjack, wet aging, physicochemical quality, metabolites

## Abstract

Wet aging is a promising strategy for improving the eating quality of high-value aquatic products, yet the metabolic basis underlying quality and flavor changes in greater amberjack (*Seriola dumerili*) remains unclear. In this study, greater amberjack was wet-aged under refrigerated conditions, and changes in physicochemical properties, taste attributes, free amino acids (FAAs), and metabolite profiles were evaluated. The pH values initially decreased and then increased, whereas moisture content declined during the aging period. Total viable counts (TVC) increased with aging time but remained below the total microbial limit for raw aquatic products. TCA-soluble peptides increased, while UFAAs increased and BFAAs and TFAAs decreased (*p* < 0.05). TBARS values also increased, indicating enhanced lipid oxidation. Electronic tongue analysis showed increased sensor responses associated with umami and saltiness after aging. Metabolomic analysis showed differences among aging stages, with glycerophospholipid metabolism, sphingolipid metabolism, and alanine, aspartate and glutamate metabolism significantly affected. During wet aging, L-glutamic acid, taste-related peptides, lipid oxidation products, and phospholipid degradation products accumulated, a pattern consistent with proteolysis and lipid remodeling. Overall, wet aging altered the instrumental taste profile and promoted the accumulation of taste-related compounds and lipid-derived metabolites that may serve as potential precursors for volatile formation in greater amberjack. These results provide the basis for optimizing wet aging for aquatic products.

## 1. Introduction

Greater amberjack (*Seriola dumerili*) is a cosmopolitan marine fish species that is highly valued by the aquaculture industry and by consumers worldwide because of its rapid growth and distinctive taste, particularly when served as sashimi [1]. The Food and Agriculture Organization of the United Nations estimates that up to 35% of global fisheries and aquaculture production is lost or wasted each year [2]. Postmortem aging is widely used in the meat industry to enhance flavor, tenderness, and palatability through endogenous enzymatic reactions [3]. Aging is generally classified as dry aging and wet aging. Dry aging requires strict control of temperature, humidity, and air circulation [4], and wet aging involves vacuum packaging followed by refrigerated storage for a defined period [5]. Wet aging has been applied to several meat products, such as beef, lamb, and pork. These products are often preferred because wet aging can enhance palatability and stability [6]. Recently, wet aging has been increasingly adopted as a strategy to modify and improve the taste of high-value aquatic products. During wet aging, endogenous enzymes promote the controlled degradation of macromolecules, especially proteins and nucleotides, into low-molecular-weight taste precursors. Taste is a key indicator of the quality of aged aquatic products, and sweetness and umami are among the most desired taste attributes. Free amino acids (FAAs) are important contributors to the quality of aged aquatic products and may also serve as biomarkers of quality changes. A previous study reported an increase in total FAAs after wet aging of tuna meat [7]. Small peptides can provide kokumi and modulate taste. Thus, taste changes during aging do not depend on a single compound class but may involve protein hydrolysis, peptide generation, amino acid transformation, and interactions among taste-active metabolites [8]. However, the dynamic changes in physicochemical quality indices and metabolites during wet aging of greater amberjack remain insufficiently characterized.

Advances in molecular sensory science and metabolomics have made it increasingly feasible to evaluate changes in food quality and flavor during fermentation, aging, and storage [9]. Electronic tongues are widely used in food taste analysis because they capture information from multiple taste-active compounds and can partially replace human sensory evaluation. Electronic tongues have already been applied to evaluate the sensory properties of fermented seafood [10,11]. However, aging-induced flavor changes are complex and are influenced by temperature, packaging, humidity, and other environmental conditions. Conventional analytical methods, such as high-performance liquid chromatography and gas chromatography, are limited in their ability to provide comprehensive qualitative and quantitative analyses of small-molecule components. Metabolomics has therefore emerged rapidly as a powerful analytical approach in recent years. Untargeted metabolomics has become an important approach for investigating metabolite variation during seafood fermentation and storage [12,13,14].

In this study, we first investigated physicochemical quality indices and then further analyzed amino acid composition and taste profiles using an electronic tongue and untargeted metabolomics. This study aimed to elucidate quality and taste changes in aged greater amberjack, thereby improving the understanding and development of aging techniques for enhancing unique sensory qualities and taste variation in aquatic products processing.

## 2. Materials and Methods

### 2.1. Fish Aging

Ten greater amberjack with an average weight of 8.0 ± 0.6 kg were obtained from a fish farm (Guangdong, China). The live fish were transported to our laboratory and euthanized by the Ikejime method [15]. The bilateral dorsal muscles of fish were excised and cut into blocks weighing 250–300 g, wiped with kitchen paper, and individually vacuum-packaged in polyethylene/polyamide bags (30 cm × 40 cm; thickness, 0.24 mm, Guangdong Jiaxin Packaging Technology Co., Ltd., Guangzhou, China). All samples were packaged using a vacuum-packaging machine with evacuation, sealing, and cooling times of 30 s, 2.6 s, and 4 s, respectively (OX-740, Ouxin Packaging Machinery Co., Ltd., Wuyi, Zhejiang, China). Ten liters of 3.5% (*w*/*w*) sodium chloride solution were prepared in a polyethylene box (44.0 cm × 33.5 cm × 18 cm) and pre-cooled at 2 °C in a low-temperature incubator (IN813C, Yamato, Chongqing, China). The vacuum-packaged fish blocks (45 packages) were then placed in the box for wet aging. At each sampling time, at least three packaged fish blocks were randomly selected for physicochemical and metabolomic analyses. A 5 mm layer was trimmed from each sample surface before analysis.

### 2.2. Determination of Physicochemical Indices

#### 2.2.1. pH Determination

A 5 g portion of fish meat was homogenized with 45 mL of ultrapure water, and pH was measured using a pH meter according to our previous method [16].

#### 2.2.2. Moisture Content

Moisture content was determined according to AOAC method No. 950.46 [17].

#### 2.2.3. Total Viable Counts (TVC)

TVC were determined as described previously [18]. Briefly, 25 g of fish meat was mixed with 225 mL of sterile normal saline (0.85%) and serially diluted 10-fold. Appropriate dilutions were mixed with plate count agar and incubated at 30 °C for 3 days.

#### 2.2.4. Color Parameter

Color was measured using a colorimeter (CM-600d, Konica Minolta, Tokyo, Japan). Three locations on each greater amberjack sample were measured by contact with the sample surface. Lightness (L*), redness (a*), and yellowness (b*) were determined by comparison with standard white and black surfaces [19].

### 2.3. TCA-Soluble Peptides

TCA-soluble peptides were determined according to the method of Wen et al. [20], with slight modifications. Fish meat (3 g) was homogenized with 27 mL of pre-cooled 5% (*w*/*v*) trichloroacetic acid (TCA) solution, kept on crushed ice for 30 min, and centrifuged (8000 rpm, 8 min, 4 °C). The concentration of TCA-soluble peptides in the supernatant was quantified using the Lowry method [21], with tyrosine used as the reference standard.

### 2.4. FAAs

FAAs were analyzed according to a previously reported method [9], with slight modifications. Fish meat (4 g) was extracted with 3% sulfosalicylic acid and homogenized at 7000 rpm for 40 s. The homogenate was centrifuged (10,000 rpm, 10 min, 4 °C), and the supernatant was mixed with 2 mL of hexane and adjusted to 50 mL with 0.02 M HCl. The solution was then filtered through a 0.22 μm membrane and analyzed using an automatic amino acid analyzer (L-8800, Hitachi High-Tech, Tokyo, Japan). Amino acid standards (Sigma-Aldrich, St. Louis, MO, USA) were used for identification and quantification by the internal standard method. The results were expressed as mg/100 g on a dry weight basis after recalculation using the measured moisture content.

### 2.5. Thiobarbituric Acid Reactive Substances (TBARS)

Fish meat (5 g) was homogenized with 50 mL of 7.5% (*w*/*v*) TCA, followed by a 30 min incubation and filtration. Subsequently, 5 mL of the filtered solution was mixed with 5 mL of 0.02 mol/L thiobarbituric acid and incubated at 90 °C for 30 min. After cooling to room temperature, the absorbance was measured at 532 nm. TBARS levels were reported as mg malondialdehyde (MDA)/kg sample [22].

### 2.6. Electronic Tongue Analysis

Instrumental taste responses of greater amberjack were measured using an electronic tongue (SA402B, Intelligent Sensor Technology, Inc., Atsugi, Kanagawa, Japan). A fish sample (1 g) was mixed with 30 mL of distilled water and homogenized to extract taste-active compounds. The homogenate was centrifuged (8000 rpm, 10 min), and the supernatant was collected for electronic-tongue analysis. The electronic tongue was equipped with sensors for sourness, astringency, umami, saltiness, and bitterness; aftertaste-astringency, aftertaste-bitterness, and richness (aftertaste-umami) were also analyzed.

### 2.7. Metabolomics Analysis

A 50 ± 5 mg portion of fish meat from each sample was combined with 400 μL of extraction solution (methanol:water, 4:1, *v*/*v*) containing an internal-standard mixture, including L-2-chlorophenylalanine (0.02 mg/mL). The sample was ground for 6 min at −10 °C and 50 Hz, ultrasonically extracted for 30 min at 5 °C and 40 kHz, and incubated at −20 °C for 30 min. After centrifugation (13,000× *g*, 15 min, 4 °C), the supernatant was collected for LC–MS/MS analysis. A pooled QC sample was prepared by mixing 20 μL of supernatant from each analytical sample and was injected once every 5–15 analytical samples. LC–MS/MS analysis was performed using a SCIEX UPLC–TripleTOF 6600 (SCIEX, Framingham, MA, USA) system equipped with an ACQUITY UPLC HSS T3 column (100 mm × 2.1 mm, 1.8 μm; Waters Corporation, Milford, MA, US). Mobile phase A was water:acetonitrile (95:5, *v*/*v*) containing 0.1% formic acid, and mobile phase B was acetonitrile:isopropanol:water (47.5:47.5:5, *v*/*v*/*v*) containing 0.1% formic acid. The gradient was 0–0.2 min, 0% B; 3.0 min, 25% B; 9.0–10.0 min, 100% B; and 10.1–12.0 min, 0% B. The column was equilibrated for 15 min before analysis. The flow rate was 0.40 mL/min, the column temperature was 45 °C, and the injection volume was 10 μL. Electrospray ionization was operated in positive and negative modes with Gas 1 and Gas 2 at 50 psi, curtain gas at 35 psi, source temperature at 500 °C, and ion-spray voltages of +5500 V and −4500 V, respectively. Data were acquired in information-dependent acquisition mode, with TOF-MS scans over *m*/*z* 50–1200 and product-ion scans over *m*/*z* 25–1200 using a collision energy of 40 ± 20 eV. Raw data were processed using Progenesis QI software (version 3.0; Waters Corporation, Milford, MA, USA) for peak identification, extraction, and alignment, then analyzed with SIMCA software (version 16.0.2; Sartorius Stedim Data Analytics AB, Umeå, Sweden) [23].

### 2.8. Statistical Analysis

Physicochemical measurements, including pH, moisture content, TVC, color, TCA-soluble peptides, and TBARS, as well as FAAs, electronic tongue, and metabolomic analyses, were based on three separately sampled fish blocks at each time point. Where means and SDs are reported, they were calculated across the three block-level values; for color, each block-level value was the mean of three within-block measurements. Univariate comparisons among aging times were performed at the block level using one-way ANOVA in SPSS 24.0. Statistical significance was assessed with Tukey‘s HSD test, considering differences significant at *p* < 0.05. Bar graphs were created using Graphpad 10.0. Metabolite variances were evaluated through PCA and PLS-DA using SIMCA 16.0.2. Seven-fold cross-validation and 200 response-label permutations were used for validation the PLS-DA. Hierarchical clustering heatmaps and pathway analysis were performed using TBtools version 1.082 (Guangdong, China) and the KEGG database.

## 3. Results and Discussion

### 3.1. pH

**Figure 1:**
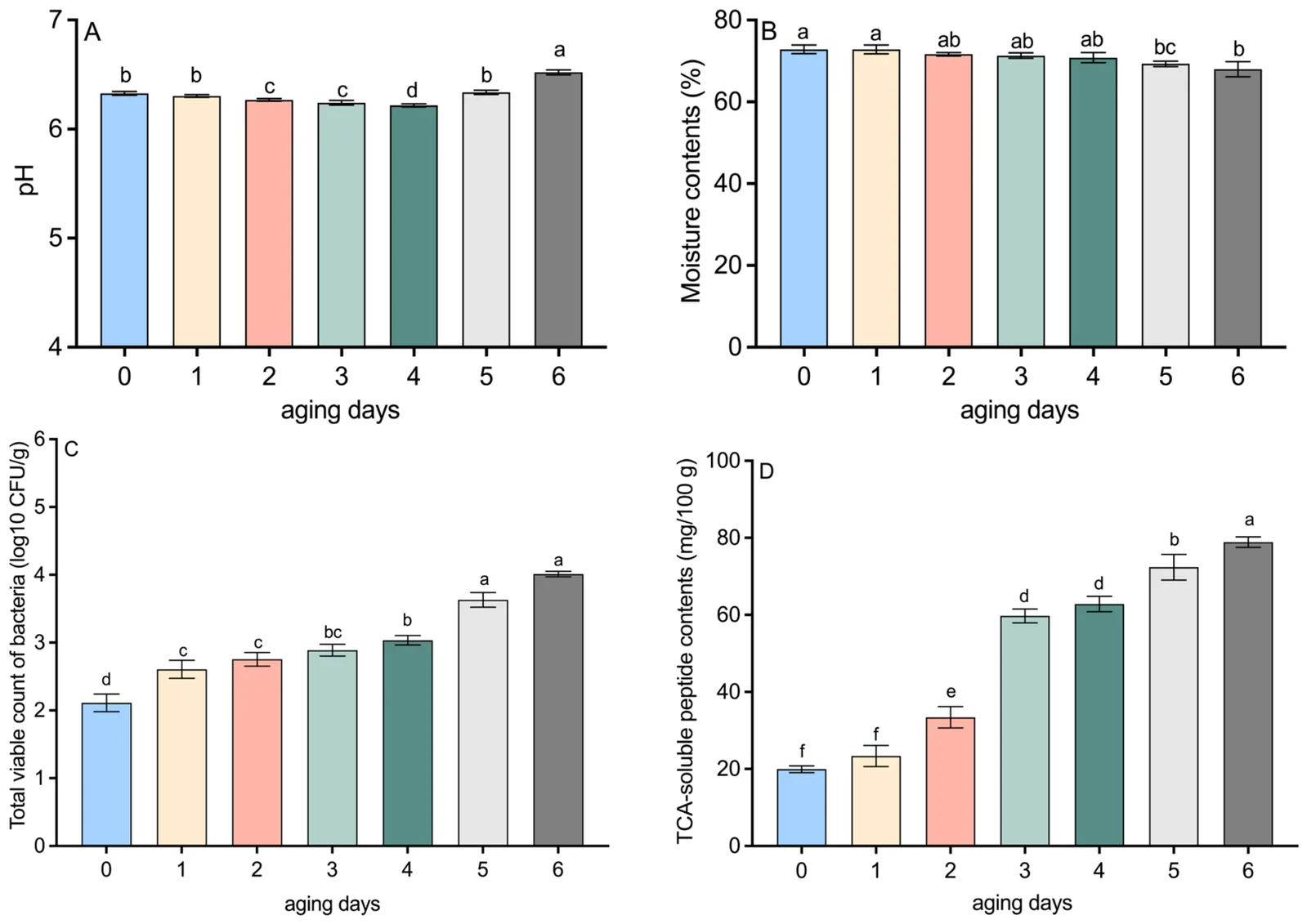
Changes in pH (**A**), moisture content (**B**), total viable counts (TVC) (**C**), and TCA-soluble peptide content (**D**) during the greater amberjack aging period. Different lowercase letters indicate significant differences among aging days (*p* < 0.05).

As shown in Figure 1A, pH initially decreased and then gradually increased as aging progressed, indicating the biochemical changes in the fish muscle microenvironment during wet aging. Similar pH fluctuation results have been reported during tuna aging, suggesting that this trend is a common feature of fish aging processes [7,24]. The early decrease in pH to day 4 may be associated with organic-acid accumulation through postmortem glycolysis, while microbial activity under the relatively anaerobic aging conditions may also have contributed [25]. During prolonged aging, pH gradually increased, which could be associated with possible accumulation of alkaline volatile compounds, including ammonia, trimethylamine, and other nitrogen-containing substances; microbial metabolism is one possible reason [24].

### 3.2. Moisture

Moisture content decreased during wet aging of greater amberjack. As shown in Figure 1B, moisture content was significantly lower after 6 days of aging than on day 0 (*p* < 0.05). This decrease may be associated with the redistribution of water from intracellular to extracellular spaces and subsequent water loss during storage [26]. Aging also induces structural changes and degradation of muscle proteins, reducing their water-holding capacity and thereby promoting moisture loss [27].

### 3.3. TVC

Total viable counts provide a general indicator of culturable aerobic microbial load [27,28]. The measured aerobic viable counts increased during aging but remained below the cited criterion for raw aquatic products. As shown in Figure 1C, the initial TVC of greater amberjack was approximately 2.11 log CFU/g and increased with aging time, reaching 4.01 log CFU/g at 6 days. This measured value remained below the cited total viable count criterion of 5 log CFU/g for raw aquatic products in China National Food Safety Standard GB 10136-2015 [29]. These results provide limited support for the microbiological acceptability of the studied wet aging period with respect to aerobic viable counts. Further safety indicators, such as pathogenic microorganisms and histamine concentrations, require additional investigation. Nevertheless, the upward trend in TVC indicates that microbial control remains a critical factor when extending aging duration. Thus, aerobic viable counts should be considered together with other relevant safety indicators and flavor improvement when optimizing aging time for industrial application.

### 3.4. TCA-Soluble Peptides

Protein hydrolysis increased steadily during wet aging. TCA-soluble peptides are commonly used as an indicator of protein degradation, and increased protein hydrolysis plays a role in taste formation in aged meat [3,30]. As shown in Figure 1D, TCA-soluble peptide content increased during aging and reached 78.91 mg/100 g after 6 days, which was significantly higher than the values at the other aging times (*p* < 0.05). The increase in TCA-soluble peptides supports progressive protein degradation during wet aging and aligns with previous studies reporting higher peptide levels during fish aging [24,31].

### 3.5. TBARS

**Figure 2:**
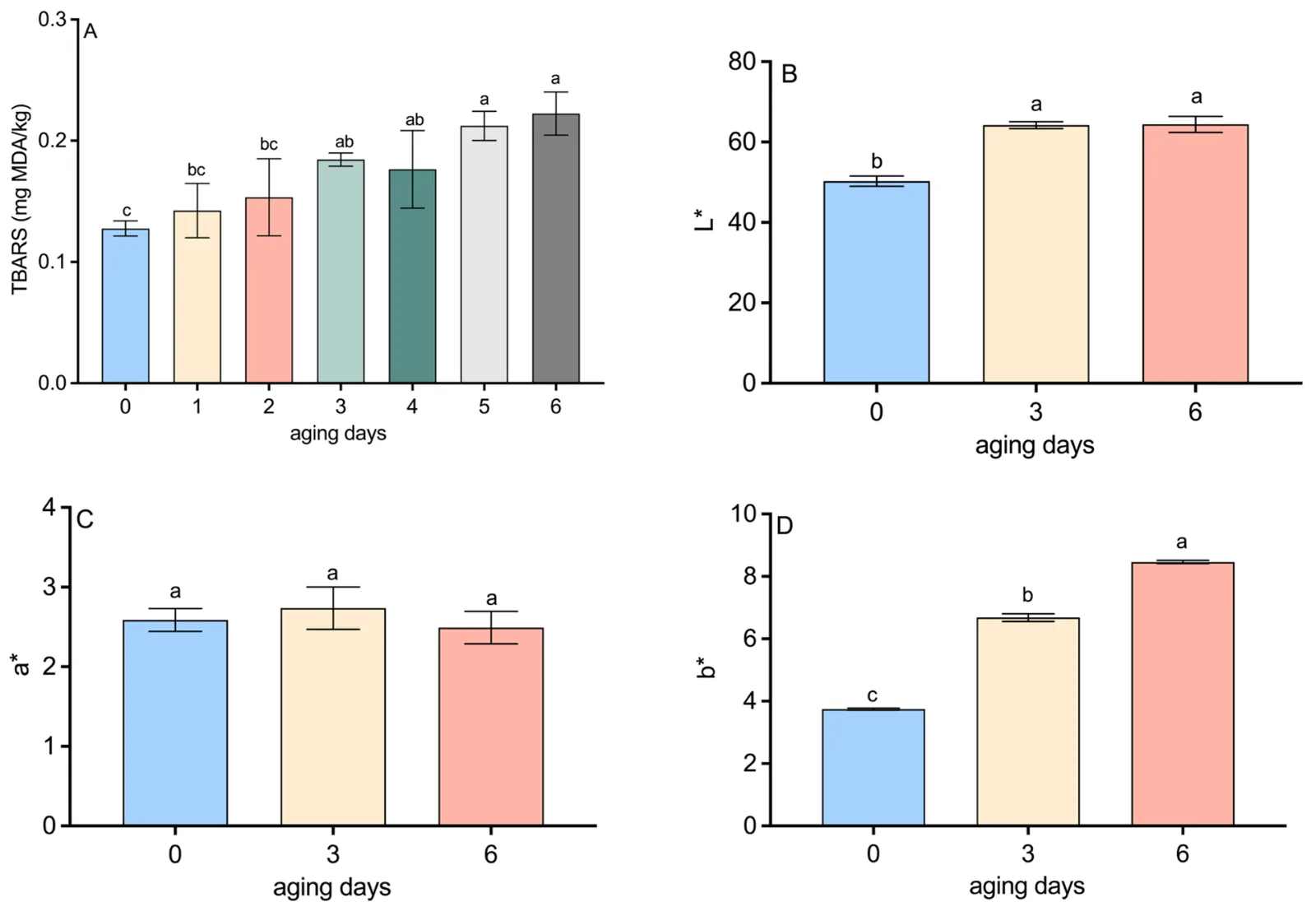
Changes in TBARS (**A**) and color parameters (**B**–**D**) during the greater amberjack aging period. L* value (**B**), a* value (**C**) and b* value (**D**). Different lowercase letters indicate significant differences among aging days (*p* < 0.05).

TBARS values increased during wet aging, indicating greater formation of thiobarbituric acid-reactive secondary oxidation products (Figure 2A) [32]. This trend suggests continued oxidation of polyunsaturated lipids during aging. Similar increases have been reported during prolonged aging and may indicate an increasing risk of oxidative quality deterioration [33,34]. Lipid oxidation may generate intermediates relevant to subsequent flavor development, whereas excessive oxidation may promote quality deterioration.

### 3.6. Color Parameters

Wet aging altered the appearance of greater amberjack as reflected by changes in the color parameters. Appearance, texture, and flavor are critical indicators of fish quality, and consumers often use visual appearance as an initial indicator of freshness and value [35]. As shown in Figure 2B–D, L*, a*, and b* values increased with aging time, and L* and b* differed significantly from those of non-aged samples (*p* < 0.05). Protein denaturation and microbial activity during storage may partly explain the increase in L* values [24,25,36]. In addition, moisture loss can enhance light reflection from the muscle surface, further contributing to increased lightness [25].

### 3.7. FAAS

**Figure 3:**
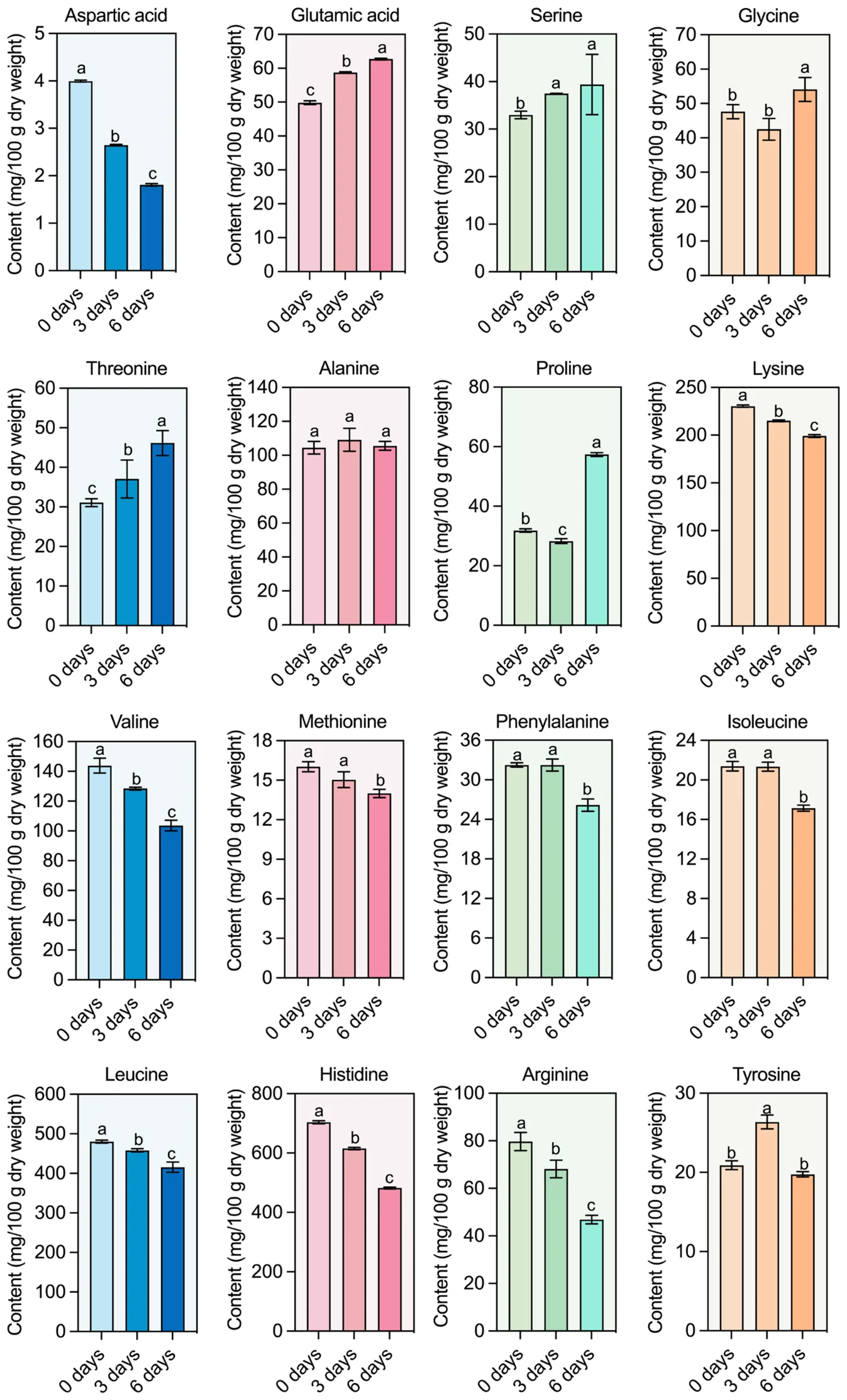
Changes in FAA contents during the greater amberjack aging period. Concentrations are expressed as mg/100 g on a dry weight basis. Different lowercase letters indicate significant differences among aging days (*p* < 0.05).

**Figure 4:**
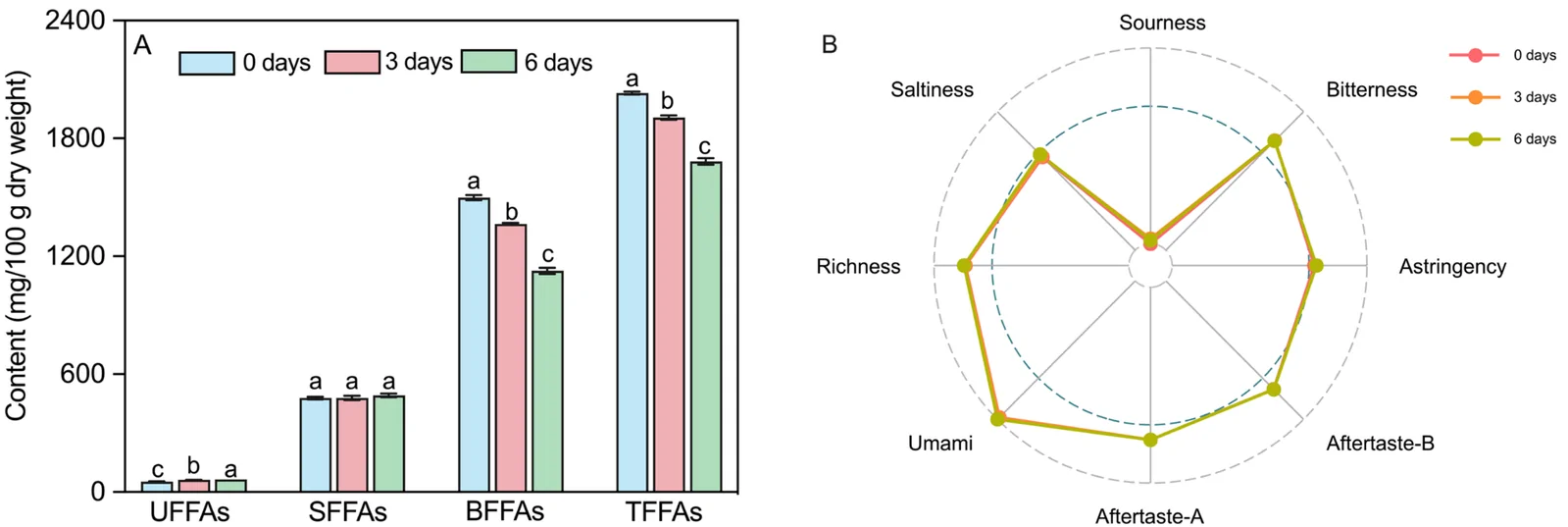
Changes in different FAA contents during the greater amberjack aging period, expressed as mg/100 g on a dry-weight basis (**A**). UFAAs represent umami free amino acids; SFAAs represent sweet free amino acids; BFAAs represent bitter free amino acids; FAAs represent free amino acids. Spider plot of electronic tongue responses associated with the taste attributes of aged greater amberjack (**B**). Different lowercase letters indicate significant differences among aging days (*p* < 0.05).

Wet aging altered the profile of taste-related FAAs in greater amberjack. Amino acids are commonly classified as sweet, umami, bitter, or other taste-related compounds [37]. As shown in Figure 3 and Figure 4A, UFAAs increased significantly during aging, whereas SFAAs showed an increasing trend (*p* < 0.05), and BFAAs and TFAAs decreased significantly. This pattern indicates a shift in the balance among taste-related FAA groups [38]. Glycine, alanine, and proline increased after 6 days of aging (Figure 3). Because glycine and alanine can mask bitterness and sulfurous notes associated with methionine, their accumulation may influence the balance among taste-related components.

### 3.8. Electronic Tongue Analysis

The electronic tongue converts chemical responses into instrumental signals associated with taste attributes and enables objective comparisons among samples [19]. Electronic-tongue analysis indicated that wet aging altered the instrumental taste profile of greater amberjack. As shown in Figure 4B, the profile changed markedly after 6 days of aging, with an increase in the response associated with umami. The sourness-associated responses of all samples were below the sensor reference threshold of −13. The saltiness-associated response of the non-aged samples was below the reference threshold of −6 but increased after aging. These results were consistent with the FAA results and indicated that wet aging altered the instrumental taste profile, particularly the responses associated with umami and saltiness.

### 3.9. Metabolic Profiles and Differential Metabolites

**Figure 5:**
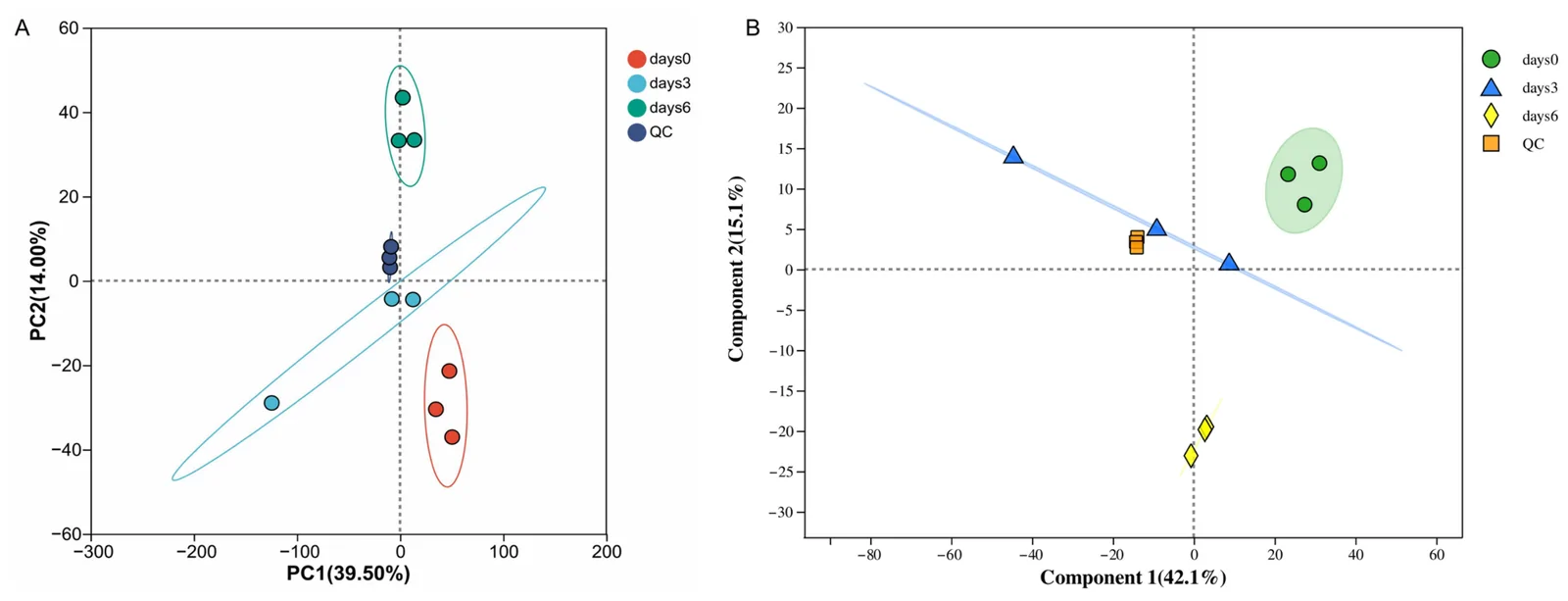
Principal component analysis (PCA) (**A**) and PLS-DA score plot (**B**) of aged greater amberjack.

**Figure 6:**
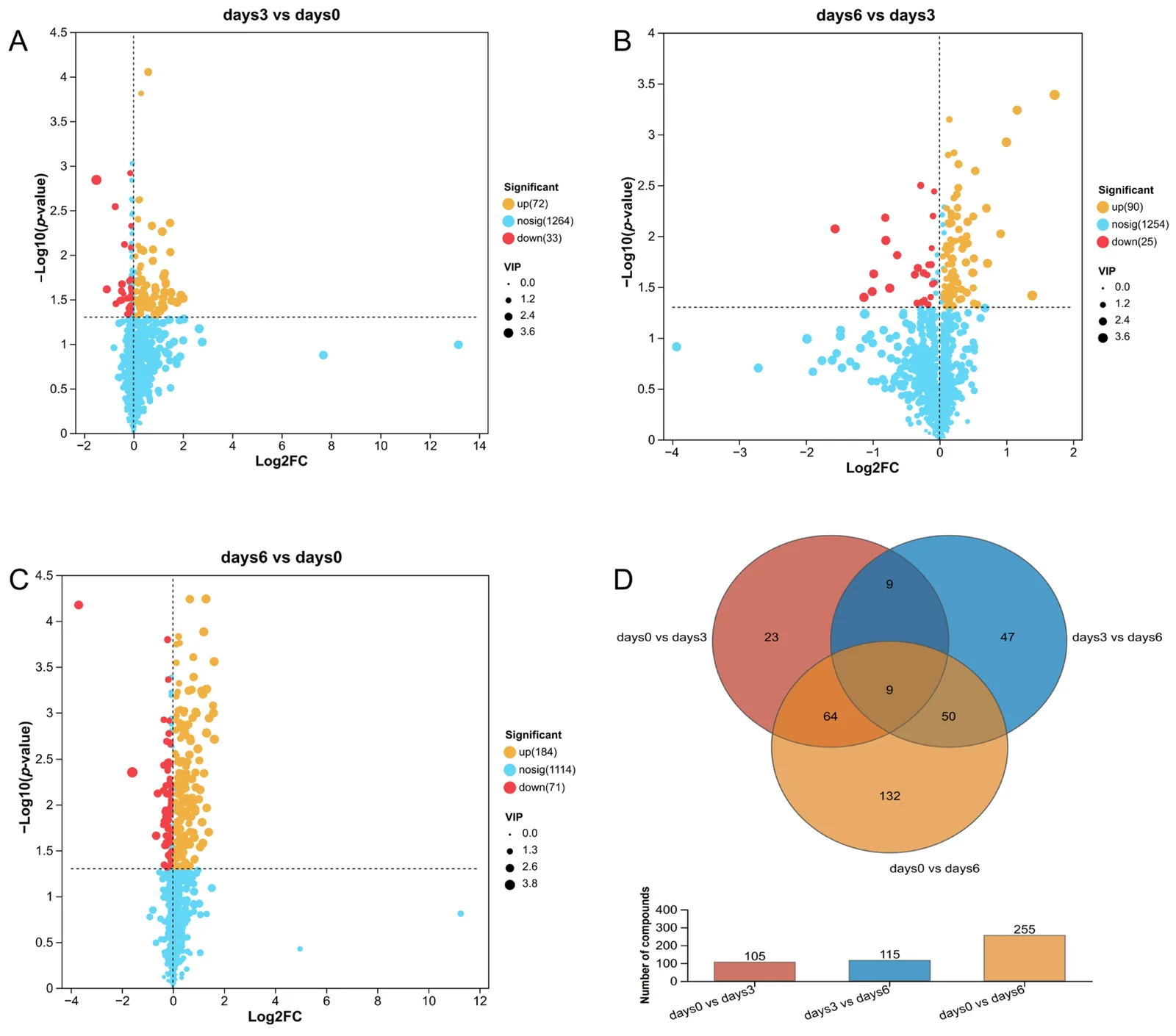
Volcano plot of the differential metabolites of day 3 vs. day 0 (**A**), day 6 vs. day 3 (**B**) and day 6 vs. day 0 (**C**); Venn plot of differential metabolites (**D**).

To comprehensively analyze the metabolic changes in greater amberjack during the aging process, we employed untargeted metabolomics to analyze the dynamics of metabolites during aging. Figure 5A shows a clear separation among the three groups, with PC1 and PC2 accounting for 39.5% and 14.0% of the total variance, respectively. PLS-DA confirmed this clear differentiation (Figure 5B), which is consistent with the PCA results. The PLS-DA model yielded R^2^Y = 0.989 and Q^2^ = 0.797. The Q^2^ intercept from the permutation test was −0.6442, supporting the absence of detectable overfitting in this validation. A total of 1756 metabolites were annotated. Using thresholds of VIP > 1 and *p* < 0.05, we identified 105, 255, and 115 differential metabolites in the day 3 vs. day 0, day 6 vs. day 0, and day 6 vs. day 3 groups, respectively (Table S1). Volcano plots showed 72 up-regulated and 33 down-regulated metabolites in day 3 vs. day 0, 184 up-regulated and 71 down-regulated metabolites in day 6 vs. day 0, and 90 up-regulated and 25 down-regulated metabolites in day 6 vs. day 3 (Figure 6A–C). As shown in Figure 6D, 23 metabolites differed between day 3 and day 0, 47 differed between day 3 and day 6, and 132 differed between day 6 and day 0. These data indicate a progressive emergence of metabolic changes during the greater amberjack wet aging period.

**Figure 7:**
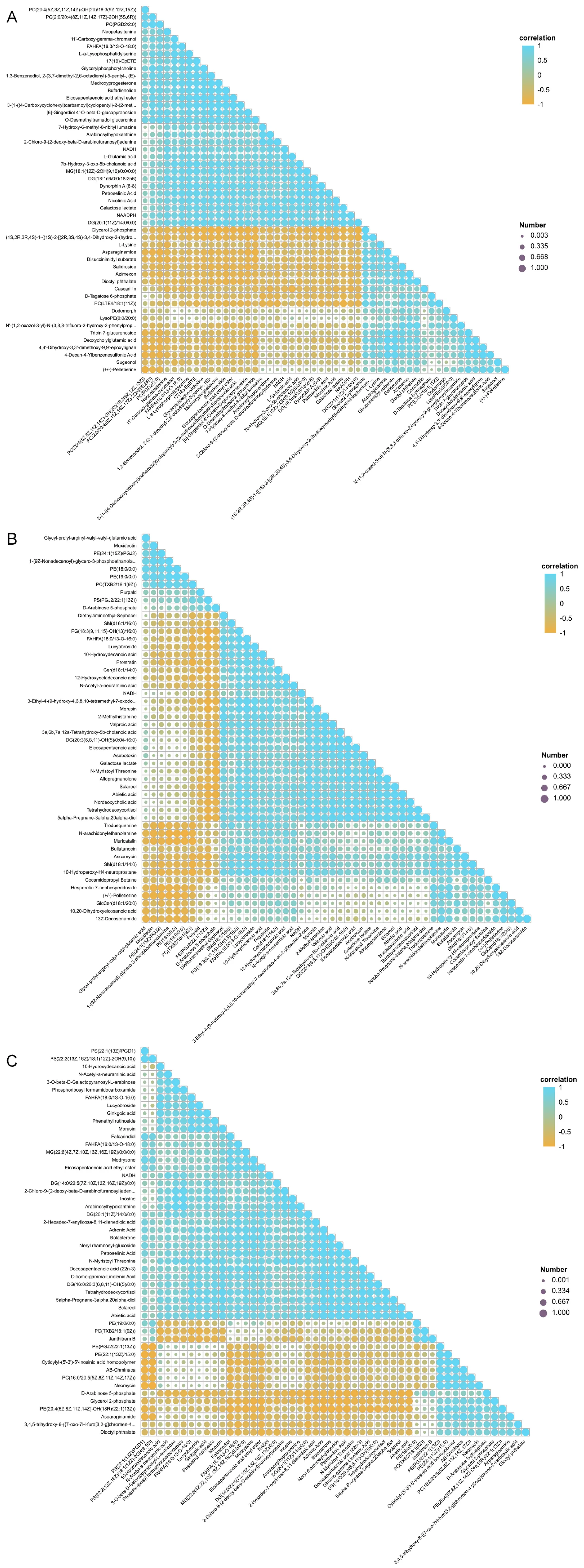
Correlation maps of the top 50 differential metabolites during wet aging, day 3 vs. day 0 (**A**), day 6 vs. day 3 (**B**) and day 6 vs. day 0 (**C**).

Figure 7 shows correlation heatmaps for the top 50 differential metabolites. In the day 3 vs. day 0 (Figure 7A), NADH and L-glutamic acid correlate negatively with glycerylphosphorylcholine and 17(18)-EpETE, while NADH and L-glutamic acid correlate positively with each other and with the flavor intermediate arabinosylhypoxanthine. These associations may be related to changes in postmortem cellular energy status, muscle autolysis, and membrane phospholipid degradation [39].

In the day 6 vs. day 3 (Figure 7B), glycyl-prolyl-arginyl-valyl-valyl-glutamic acid and lipid auto-oxidation products (12-hydroxyoctadecanoic acid and 10-hydroperoxy-H4-neuroprostane) correlate positively with intact membrane lipids PC(TXB2/18:1(9Z)) and SM(d16:1/16:0). Concurrent structural lipid alterations, proteolysis, and lipid oxidation may arise from progressive disruption of lipid bilayers, which permits enzymatic leakage, thereby promoting proteolysis and exposing liberated lipids to oxidation [39,40].

In the day 6 vs. day 0 (Figure 7C), inosine, adrenic acid, and 10-hydroxydecanoic acid are consistently positively correlated with each other and with eicosapentaenoic acid ethyl ester. These compounds are negatively correlated with N-acetyl-α-neuraminic acid and with intact lipid derivatives such as morusin and falcarindiol. Correlations between inosine and fatty acid esters suggest these metabolites may be linked to flavor changes during aging, but their roles in volatile formation and in perceived taste require further evaluation.

**Figure 8:**
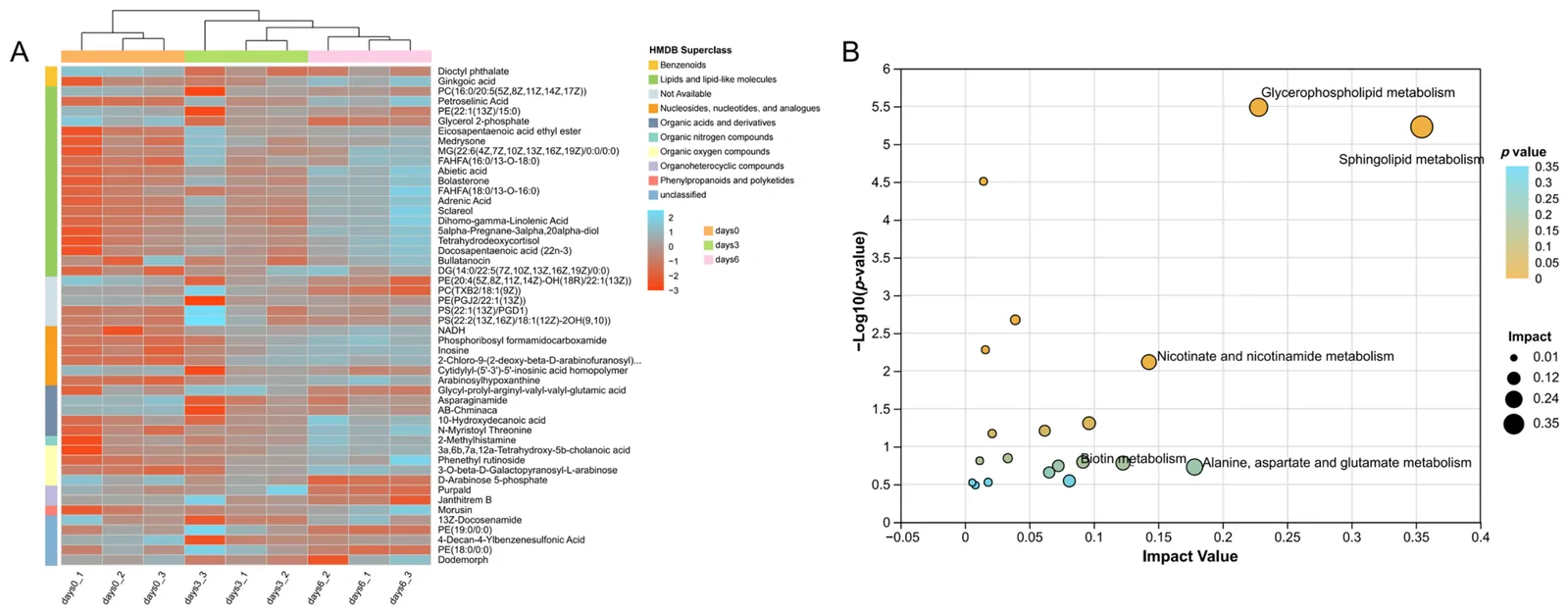
Heatmap of hierarchical cluster analysis of significantly different metabolites (top 50) during the greater amberjack aging period (**A**); Bubble plot of KEGG metabolic pathway enrichment analysis of aged greater amberjack (**B**).

To compare metabolite composition across groups, we performed hierarchical clustering on the top 50 differential metabolites. Figure 8A shows the resulting metabolite profiles for the aging groups. The clustering showed greater within-group similarity and greater variability between groups. KEGG pathway analysis identified lipid and amino acid metabolism as the primary pathways altered by wet aging. The top 20 enriched KEGG pathways included glycerophospholipid metabolism, sphingolipid metabolism, and alanine, aspartate and glutamate metabolism (Figure 8B). These enrichment results were associated with metabolite changes potentially related to membrane lipid remodeling, lipid oxidation, and amino-acid-derived taste-precursor formation. Consistent with this interpretation, lipids and lipid-like molecules were the largest annotated superclass in the metabolite class distribution (Table S1), especially in day 6 vs. day 0. Enrichment of glycerophospholipid and sphingolipid metabolism is notable because phospholipids are major muscle cell membrane constituents; their postmortem enzymatic hydrolysis and oxidation could result in the release of fatty acids and lysophospholipids that may serve as substrates for subsequent volatile formation. Concurrent changes in metabolites related to alanine, aspartate and glutamate metabolism were observed alongside the rise in umami-related compounds, which may link pathway-level changes to the measured taste data; however, further studies are needed to support this interpretation.

Amino acids and their derivatives directly participate in protein degradation, taste formation, and flavor conversion [41]. L-glutamic acid increased during aging, indicating proteolysis-mediated release of UFAAs. In contrast, lysine and asparaginamide decreased, consistent with the FAAs results. These changes may reflect both amino acid release and subsequent conversion or utilization, potentially involving endogenous enzymatic and microbial activity [42].

Peptide accumulation further increased the diversity of taste-related compounds during wet aging. Peptide-related compounds, including γ-glutamylfelinylglycine, dynorphin A (6–8), and Gly-Pro-Gly-Arg-Ala-Phe, increased during aging, whereas Mucronine D and Trp-Pro-Trp decreased from day 3 to day 6. The accumulation of γ-glutamylfelinylglycine suggests an increase in γ-glutamyl peptides, which have been associated with kokumi characteristics [43]. Meanwhile, the increased levels of dynorphin A (6–8) and Gly-Pro-Gly-Arg-Ala-Phe, particularly peptides containing Pro, Arg, and Phe residues, may be associated with bitterness. However, the bitterness-associated electronic-tongue response decreased, whereas UFAAs and SFAAs increased, and BFAAs decreased. These changes may have contributed to the observed instrumental taste profile. A similar phenomenon has been reported during mandarin fish fermentation, in which several bitter peptides increased but did not negatively affect overall taste because freshness-related amino acids reached high levels [10].

Lipid degradation and oxidation may contribute to the formation of aroma-related compounds in seafood [9]. After 3 days of aging, increases in 9-hydroxy-10,12-octadecadienoic acid and eicosapentaenoic acid derivatives suggested the initiation of lipid hydrolysis and mild oxidation [44]. After 6 days of aging, lipid metabolism became more active, as reflected by increased methyl linolenate, 20-hydroxyeicosatetraenoic acid, undecylenic acid, eicosapentaenoic acid, and other lipid oxidation-related products. This trend was consistent with the TBARS results, suggesting intensified lipid oxidation during the later aging stage. In addition, increased phospholipid-degradation products (phosphatidylcholine-related products, lysophosphatidic acid-related products and glycerylphosphorylcholine-related products) supported membrane lipid remodeling, consistent with enrichment of glycerophospholipid and sphingolipid metabolism. These reactions may generate potential precursors for subsequent volatile formation, but excessive oxidation can produce rancid notes and reduced nutritional quality. Therefore, lipid oxidation may contribute to flavor development and may also be a potential limiting factor for prolonged wet aging.

Overall, the observed increases in TCA-soluble peptides and specific taste-related amino acids during early wet aging were consistent with possible endogenous proteolysis; these amino acids included glutamic acid, which is associated with umami and kokumi characteristics. Meanwhile, changes in lipid-derived intermediates were consistent with possible initial lipid hydrolysis and mild oxidation, and these intermediates may serve as substrates for subsequent volatile formation. With prolonged aging, continued proteolysis was associated with greater peptide diversity and a broader range of taste-related compounds, whereas changes in phospholipid-related metabolites and TBARS were consistent with possible phospholipid remodeling and fatty-acid oxidation. These changes are reflected in the larger number of differential metabolites and elevated TBARS values observed in day 6 samples. However, although extended aging may provide additional potential flavor-related intermediates, it may also be accompanied by increased risks of quality deterioration associated with lipid oxidation and microbial growth.

## 4. Conclusions

This study systematically characterized the physicochemical and metabolomic changes occurring in greater amberjack during wet aging. Wet aging was accompanied by increased TCA-soluble peptides and an altered taste-related FAA profile, including increased UFAAs and decreased BFAAs. These changes occurred alongside increased umami-associated electronic tongue responses and greater diversity of taste-related compounds. Untargeted metabolomics further identified changes in metabolites related to amino acid metabolism, peptide accumulation, glycerophospholipid and sphingolipid metabolism, and lipid oxidation; some of these metabolites may serve as potential precursors for subsequent volatile formation. Our results indicate that wet aging altered the instrumental taste profile and flavor-related metabolite composition of greater amberjack, while emphasizing the need to optimize aging duration. These results provide new insights into potential biochemical processes associated with wet aging of aquatic products and offer a scientific basis for developing wet-aging strategies that balance flavor-related changes with product quality and shelf-life requirements. Future studies integrating volatile compound profiling, microbial community analysis, and multi-omics are needed to further investigate the potential mechanisms of wet aging in aquatic products and optimize its industrial applications.

## Data Availability

Data will be made available on request.

## Supplementary Materials

The following supporting information can be downloaded at: https://www.mdpi.com/article/10.3390/foods15183311/s1; Table S1: A complete list of differential metabolites.
